# A Process-Centered Approach to the Description of Clinical Pathways—Forms and Determinants

**DOI:** 10.3390/ijerph16152638

**Published:** 2019-07-24

**Authors:** Marek Szelągowski, Justyna Berniak-Woźny

**Affiliations:** 1 Systems Research Institute, Polish Academy of Sciences, 00-001 Warsaw, Poland; 2 Faculty of Business and International Relations, Vistula University, 02-787 Warsaw, Poland

**Keywords:** business process management, dynamic business process management, clinical pathways, knowledge management

## Abstract

The aim of the study is to indicate the need for variability in the presentation of clinical pathways, in various phases of their implementation, and to define the forms of presentation of clinical pathways required by physicians in both the Hospital Information Systems (HIS) and Electronic Medical Records (EMR) Systems, as well as the determinants of the selection of the forms of description, in relation to the performed medical actions. The results of the study are a significant lead-in towards further research on the required form of the user interface in systems supporting dynamic business process management (dynamic BPM). The research is a pilot of a survey study, conducted to ascertain the usefulness and feasibility of the adopted methodology, for a wider project on the determinants of the form of description of clinical pathways. An exploratory pilot survey, in a large multi-specialization hospital in Poland, was conducted. The survey sample consisted of 28 purposely selected heads of all hospital departments, and the medical team of the pediatric ward. Descriptive analysis was carried out on the data collected. The results of the study have unambiguously supported the claim that physicians require the form of presentation of clinical pathways to change, depending on the particular phase of the diagnostic–therapeutic process, as well as establishing the main determinants thereof. This pilot study is one of the first attempts to establish the factors determining the choice of clinical pathway presentation in HIS/EMR systems. While not conclusively decisive in terms of the forms of presentation or the determinants of their choice, it indicates the directions of further research, both from the point of view of ergonomics and the usability of HIS/EMR systems, as well as the management of medical knowledge, as part of the dynamic management of clinical pathways.

## 1. Introduction

Modern management offers a broad range of methodologies, tools, and technologies which facilitate ongoing operations and raise the efficiency of the decision-making process and performed actions [1,2,3,4,5]. Such solutions are increasingly used, more often and more broadly, in the field of healthcare. Their implementation results in raising the efficiency and quality of the performance of diagnostic and therapeutic processes, their cost optimization, time reduction, and reduction of used resources [6,7,8,9,10]. Due to their dynamic character, and often their unpredictable nature, this necessitates the synthesis of what would seem to be mutually exclusive requirements—administration and supervision priorities, as well as physicians’ requirements (Table 1).

One of the ways of achieving the aforementioned goals is the use of process management in healthcare [9,11,12]. The initial phase usually consists of attempts at describing patient treatment from a process-based perspective. This is achieved through the identification of diagnostic and therapeutic processes called clinical pathways (CPs), which are, in essence, the standard procedure in a given healthcare unit. Unfortunately, in most cases, the use of process management in healthcare is limited to preparing diagrams with models of CPs. This goes against the principles of process management, which stresses the importance of performing comprehensive actions, which would harmonically combine management philosophy, methodologies, and process management tools [13,14] with the aim of directing processes toward a shared, comprehensive goal (in order to avoid sub-optimization), engaging, and making responsible, all of the participants in the process, and the ongoing optimization of processes. In effect, when implementing process management in healthcare, one should not forget that:

- It is the patient, not the disease, who is being treated;
- The treatment process should include the patient and their closest relatives;
- Different clinical and extra-clinical pathways (management, support, logistics, and others) should comprise a cohesive system;
- Clinical pathways should be reflected in IT systems, used on an ongoing basis by physicians and other relevant personnel.

Another goal that is tied to performing processes in healthcare is the management of knowledge used in planning treatments and treating patients. From this perspective, CPs should perform the role of a repository of knowledge and a model against which both current and new knowledge from patient treatment is verified [12].

The preparation of CP models is an essential part, albeit just one of multiple parts, of implementing process management in healthcare. We cannot expect measurable results if the identification of CPs marks the end of the entire implementation. It is essential to ensure that the knowledge contained in CPS be accessible in the course of treatment, and that clinical decisions and therapeutic steps be freely made on an ongoing basis, as well as to ensure that the results of using existing knowledge (steps in accordance with the standard clinical pathway) and the creation of new knowledge (steps deviating from the standard clinical pathway) are analyzed and evaluated [15]. In terms of the Hospital Information Systems (HIS) and Electronic Medical Records (EMR) systems supporting physicians, this requires the transparency of the form of presentation of knowledge, including by the patient’s bedside, as well as the ergonomic, simple form of inputting information and observations on an ongoing basis by the physicians. The reason for conducting the research presented in the article is the gap between detailed modeling of CPs, and the possibility of their use and enrichment, limited by the failure to include in the HIS and EMR Systems, the possibility of presenting or entering data in a manner consistent with the physicians’ requirements, and hence dependent on the implementation phase of CPs.

This paper contributes to the literature on the adoption of business process management (BPM) in healthcare organizations. As noted by González Sánchez et al. [16], while BPM in healthcare has only been used in the last two decades, it has been proven, however, that “this structured approach to clinical work can lead to relevant cost reductions and better outcomes, but most of all, can provide leverage for clinical process control, as well as the possibility to study the gaps between standard protocols and specific clinical complexities” [17]. For example, the study of the use of BPM in the management of kidney transplantation provided genuine benefits in terms of resources optimization and quality improvement. The percentage of time saved by using the new method was just short of 60%, laboratory time reduction was nearly 40%, time reduction with respect to planning subsequent admissions was 100%, and time reduction with respect to communicating plans to the stakeholders within the department was 70%. This study also proved that there was a reduction of human errors due to the automatic management of admission appointments and the associated protocols and automatic e-mail planning for the nurses. This means that the quality of data and the reliability of the pathway planning have been improved [17,18].

Structurally, this article is organized as follows: first, the related works section will provide an overview of process management and a clinical pathway in general, along with a process-based approach to describing CPs. The second part outlines the methodology employed in this study and the justification of the sample. The research results are presented next in the Results and Discussion sections. Finally, the authors conclude with a summary of findings, limitations of the study, and some ideas for future research.

## 2. Related Works

The aim of the section is to define the conceptual framework of the study, as well as the principles of adopting process-centered methodologies in the field of healthcare.

### 2.1. Business Processes

The concept of processes and process management itself stems from production management [19]. Initially, processes were understood as sequences of actions that needed to be performed in accordance with a pre-defined description (production manual, procedure, rules and regulations, models, algorithms), with a view to achieving an expected result (product). Most definitions of a traditional business process refer to the following characteristics: predictable and definable inputs; a linear, logical sequence or flow; a set of clearly definable and inter-related activities; predictable and desired outputs [13,20]. Palmberg [21] also adds: horizontal or cross-functional; performed with the use of resources; repeatable, and adding value for customers and stakeholders.

The aim of traditional process management was to design and implement, in production, the optimized (or best) process out of the processes available at hand, and subsequently, to monitor whether work is going according to plan [22,23]. Processes were designed, executed, and periodically improved on the basis of the knowledge of the designer or the design team instead of the knowledge of the process performers, available in the design phase, instead of in the course of execution itself. This approach is only feasible when the process does not undergo changes or changes in periods which are much longer than the length of execution. Such processes include processes defined by law like accounting or financial ones; internal processes defined by organizations (e.g., settlement of invoices, acceptance of holidays); production processes highly adapted to objective external criteria (e.g., biological and chemical), or held patents and licenses.

This approach was, and still remains, justified in the case of processes, for which it is reasonable to detach process evaluation and design (thinking) from execution itself (doing). This is logical, as, for instance, it is impossible for each car engine to have a different piston diameter, or for each accountant to make use of creative bookkeeping principles. The type of managed processes (repeatable production processes) and the main requirements (ensuring constant product quality) have determined the form of description and management. However, with the accelerating pace of changes to products and services, the understanding and definition of the process have begun to change as well. In accordance with the works of Michael Porter [24] on the value chain (1985), it was accepted that business processes encompass not just the production phase, but the entire operations of an organization, including service-oriented organizations. This, in turn, has resulted in changing the approach to the business process. It has been noted that business processes often pertain to co-ordination with a view to achieving goals, rather than to a pre-defined and strict definition of a sequence of tasks, which need to be accomplished [13,25,26,27]. In the case of service-oriented processes, it is often particularly hard to describe, and then expect adherence to a detailed performance of tasks, pre-defined in the form of a procedure, or rules and regulations. As the expectations, habits, or the limitations of the clients or the context of a specific performance differ in each circumstance, or are even mutually exclusive, the key to success is not to execute an optimal or ‘ideal’ process developed in the course of an—e.g., periodic organizational Continuous Process Improvement (CPI)—but rather, to competently and dynamically shape business processes, in accordance with client expectations, and the specific context of performance, by taking account of the tacit knowledge of the process performers themselves. Depending on the type of the process, this may require the empowerment of the process performers to undertake ad hoc actions in the course of performance itself, within a pre-defined process structure, or simply structuring only pre-defined fragments or fundamental milestones of process performance [27,28]. 

**Figure 1:**
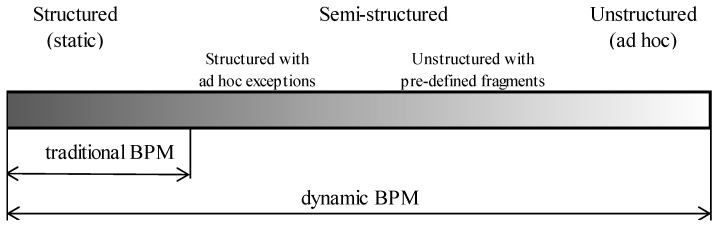
The degree of business processes structurization. Source: Authors’ own framework, on the basis of [12,27,31]. BPM: business process management.

The implementation of dynamic BPM should be performed in such a manner that the execution of a process will match its documentation, including the documentation of all changes and innovations [29,30]. Based on the Kemsley research [31], due to the dynamics of implementation processes, can be divided into (Figure 1):

- Structured (static, predictable, repetitive) processes—as described above, processes which can be described in detail, in advance, and optimized, due to the pre-defined conditions of all decisions taken at the time of implementation;
- Semi-structured processes, further divided into:
  - Structured processes with ad hoc exceptions—processes for which detailed description is possible before the commencement of implementation, and determination of decisions, as a result of which, ad hoc individual tasks not provided for in the process description, can be realized.
  - Unstructured processes with pre-defined fragments—processes, for which it is possible, to clearly define goals and the roles of participants, and to describe in detail, the fragments, with which implementation must comply, within the imposed standards.
- Unstructured (unpredictable) processes, where it is possible to define the aims of the process, but is impossible to define as a priority, the exact steps to be taken, in order to successfully execute the process.

In each case, this requires empowering employees to introduce adaptations to the process in the course of the performance, in accordance with their level of privileges, and their entire body of knowledge, not just explicit knowledge [32]. 

The definition of a business process cannot be limited to a business process understood as a pre-defined sequence of actions [29,33]. It must be naturally expanded to encompass processes, the course of which cannot be foreseen in detail, for which only goals, indicators, available resources, or other attributes can be defined [34]. This requires the de facto extension of traditional, static process management, with a view to managing unstructured or semi-structured processes. One proposal of such an extension is dynamic BPM, understood as an extension of traditional process management, which empowers process performers to introduce dynamic adaptations to the requirements of a specific performance in the course of the actual performance in accordance with the following three principles [35]:

- The first principle: comprehensiveness and continuity;
- The second principle: process execution should guarantee evolutionary flexibility;
- The third principle: processes are considered completed, only after having been documented.

The first principle assumes that management, in accordance with dynamic BPM, is a holistic approach, which must be centered on the overall goal of the process, from the perspective of the client (e.g., improving or maintaining the patient’s health, not just curing a specific disease). The second principle assumes that in order to achieve this goal, process performers must be allowed to make use of their entire body of knowledge and their dynamism, including being able to dynamically adapt the performed process to the context of a specific performance, in accordance with their level of privileges. The third principle assumes that the implementation of dynamic BPM should be performed in such a way as to ensure that processes are considered completed only after having been documented, including the documentation of all introduced changes and improvements [29,30,35]. As highlighted by Czekaj [36], “Dynamic business process management is not just an extension of the classical concept of process management, but also an attempt to harmonise process management with the concept of the learning organisation. This is achieved through the ongoing verification of acquired knowledge, with respect to the needs of the clients by numerous process performers, which leads to the gradual accumulation and proliferation of such knowledge”. In the case of dynamic BPM, it is the knowledge of the process performers which determines the execution of a process in a specific context. One particular type of dynamically managed processes are knowledge-intensive business processes (kiBP), which are capable of generating expected value only by using the knowledge of the process performers themselves, performing inter-related tasks requiring the making of decisions on the basis of their entire knowledge, both tacit and explicit [37,38].

Due to the nature of diagnostic and therapeutic processes, it is impossible to describe, execute, and perform ex-post evaluation thereof, in accordance with the principles of traditional BPM [12,35,39]. For this reason, such processes were initially managed not on the basis of process-centered tools and methodologies, but tools and methodologies derived from case management. They enabled the management of unstructured processes, for which it was impossible to prepare a detailed process pattern. They allowed the definition of actions which may be performed depending on the decisions of the knowledge worker executing the process, and enabled the collection of information on process performance [28,40]. For this reason, one of the initial names of plans for diagnostic and therapeutic processes was “case management plans” [41]. Even as recently as in 2012, it was still broadly believed that dynamic (unstructured, unpredictable, ad hoc) processes, including diagnostic–therapeutic processes, could not be modeled at all, which also means they cannot be managed [27]. It is only following the extension of traditional process management to dynamic BPM, that it became possible to manage unstructured processes, or even processes which are completely unpredictable [27,42]. This has allowed us to bridge the artificial gap between case management and process management [35]. When adopted in healthcare, dynamic BPM allows for the preparation of process descriptions for diagnostic–therapeutic processes, the support of their performance, and the collection of data on the actual course of the treatment, as well as allows for the analysis of variances of the standard clinical pathway, at any point in its course [34]. In accordance with the first principle, dynamic BPM still requires an efficient, structured format for recording key clinical data in case notes [15,43], which are CPs.

### 2.2. Clinical Pathways as Processes

Clinical pathways (CPs), initially named case management plans—and also known as care pathways, critical pathways, integrated care pathways, coordinated care pathways, care maps, or anticipated recovery pathways—are all forms of a term with a definition, which is still in the process of being formulated. De Bleser [44] notes that a total of 84 different definitions of pathways have been described and used in publications [8,15,39,45,46,47]. The European Pathway Association (EPA) defines a care pathway as a complex intervention for mutual decision making and organization of care processes for a well-defined group of patients, during a well-defined period [48,49,50]. With regard to the above-mentioned definition of a process, a “clinical pathway” should be understood as a dynamically managed diagnostic–therapeutic process describing the inter-related decisions and actions made and undertaken with a view to improving or maintaining the health of the patient. The aim of a CP is to enhance the quality of care measured by improving risk-adjusted patient outcomes, promoting patient safety, increasing patient satisfaction, and optimizing the use of resources [50]. According to Wolff et al. [51], critical success factors (CSFs) contributing to successful development and implementation of the clinical pathway include, among others:

- Analysis of related works in order to determine the best clinical practice for each medical condition and incorporating it into the CPs;
- Definition of the care process in each CP;
- Creation of the multi-disciplinary teams and granting ownership of each pathway disciplines involved in the care process;
- Invitation of all medical professions to comment on each pathway before their implementation;
- Incorporation of CPs into the patients’ medical records;
- Implementation of the regular feedback loop to all health professionals involved in the CP.

Not only are pathways a document in the patient’s record, but also a way to organize and standardize multi-disciplinary care for patient groups, using well-known quality improvement methods [47,52]. CPs do not only take the form of a diagram or a description of a planned treatment (e.g., an Individual Treatment Plan—ITP), but ideally, the integrated care pathway should incorporate medical notes, together with care plans of doctors, nursing staff, and of professionals allied to medicine [15]. Reports of the Joint Commission on Accreditation of Health Care Organizations [53] show that 70% of medical errors are caused by a lack of proper communication between team members. In effect, the form of presentation for CPs should be tailored to the transparent exchange of information between different members of inter-disciplinary teams responsible for patient treatment, in different phases of the planned treatment process, as well as to the ongoing entry of data on the treatment and its modifications [12].

**Figure 2:**
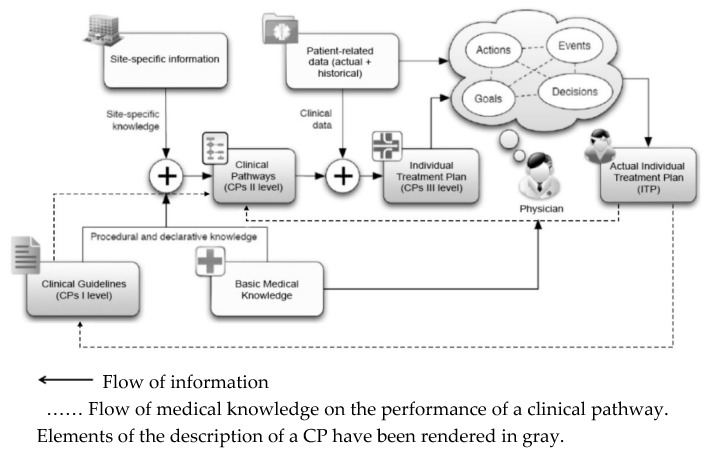
The flow of information from Clinical Guidelines (first level CPs) to the actual Individual Treatment Plan (third level CPs). Source: Authors’ own framework, based on [12]. CPs: clinical pathways.

**Figure 3:**
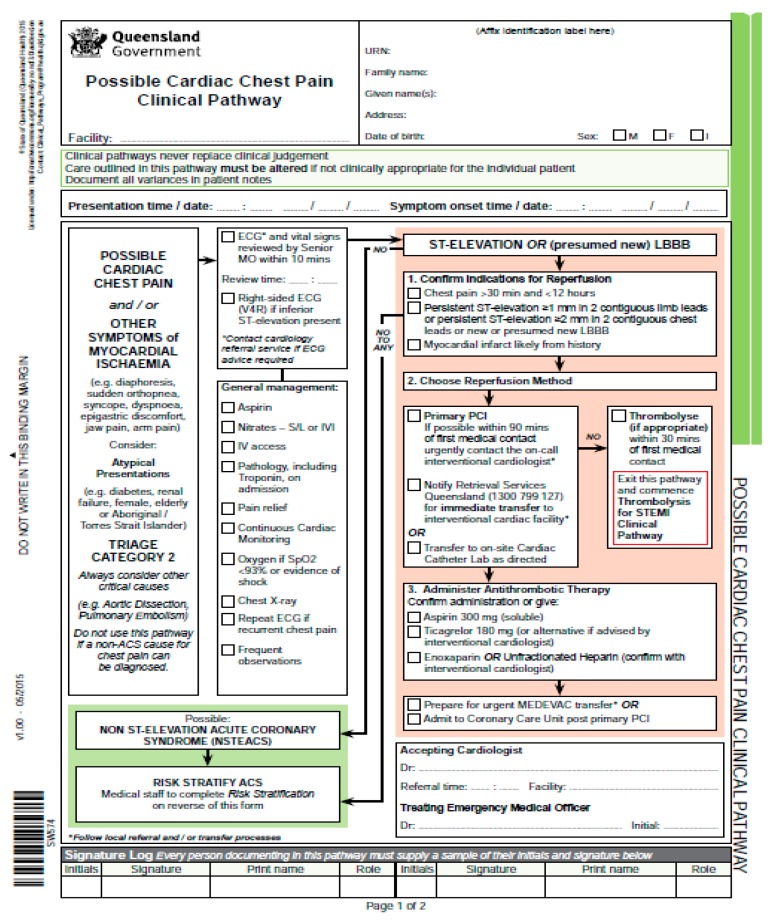
Example of the clinical pathway: “Possible Cardiac Chest Pain Clinical Pathway”. Source: State of Queensland [60].

However, as the schematic diagram in Figure 2 demonstrates, the preparation of a CP in the form of an Individual Treatment Plan (ITP) cannot be detached from the broader process of distributing and updating medical knowledge. The actual beginning of the creation of a CP rests in the preparation of international or national guidelines on the treatment of specific diseases (first level CPs) by teams of experts on the basis of current medical knowledge. On this basis, with limitations in mind, with respect to available hardware and material resources, as well as the knowledge and skills of the medical staff, particular healthcare units then prepare binding clinical pathways (second level CPs). Only on this basis do physicians prepare third level CPs for individual patients, in the form of ITPs [54]. When implemented in systems supporting the work of physicians and other medical personnel (e.g., HIS or EMR), these plans allow for the presentation of the planned and performed treatment, and, first and foremost, the entry of current data on patient treatment, including ITP adaptations and upgrades, in accordance with the requirements of a specific treatment. The analysis of data collected in the course of a clinical pathway, enables constant improvements, with respect to the efficiency and quality, as well as the economic efficiency of the treatments [43,47,55,56]. As the dashed green lines on Figure 3 indicate, it also allows for the constant improvement of first and second level CPs.

In effect, CPs perform the function of not just dynamically managed diagnostic–therapeutic processes, but at the same time are a crucial element of the distribution, verification, collection, and creation of new medical knowledge. The distribution of knowledge is of particular significance, and for this reason, it should encompass all of the members of the diagnostic–therapeutic process. In effect, apart from CPs dedicated to physicians and medical personnel, particular focus should be given to the development of CPs dedicated to [45,49]:

- Patients and their closest relatives,
- Students of medicine and inexperienced doctors making first steps in a given speciality.

Thanks to new technologies from the fields of IT and robotics, such as process mining, machine learning, artificial intelligence, and predictive analytics, in accordance with the third principle of dynamic BPM, it should be possible to document processes in the course of the performance, itself, without the necessity of burdening doctors with additional work. However, this requires the HIS/EMR systems supporting the work of physicians and the remaining medical personnel to meet the following three conditions:

1. Presenting the planned and performed CPs in a way that is tailored to the requirements of the performers;
2. Possibility to present and enter data in the place where the clinical pathway is being executed, that is, at the patient’s bedside, in a clinic, ER, ambulance, or even in the patient’s home;
3. An ergonomic approach to data entry, which is cognizant of the limitations faced at each specific phase of the process, with the use of robotic process automation and elements of artificial intelligence, such as voice recognition, image recognition, and recognition of handwriting.

As early as in 1989, Campbell [15] suggested that integrated care pathways, so far largely hospital-based, will continue to develop and be extended into primary care, rehabilitation, and community settings. At present, within the concept of coordinated care there is often a return to attempts at a comprehensive description of CPs as an integrated treatment, planned and performed from the point of view of the patient’s care, not just a particular CP for one isolated healthcare unit [57].

### 2.3. Analysis of Existing Forms of Describing Clinical Pathways

As highlighted by Cheah [58], CPs are multidisciplinary care plans used by healthcare professionals as a guide to concurrently plan, coordinate, deliver, monitor, document, and review care. CPs are continuously updated and reviewed (by a group of physicians, nurses, and other health professionals) becoming a method for evaluating the care provided and important component of continuous quality improvement (CQI) in clinical practice. As the consequence of this multidisciplinary and dynamic nature of the diagnostic and therapeutic processes, it is impossible to define and use CPs in the form of a treatment algorithm, the performance of which would guarantee patient recovery. What is more, the execution of CPs highly dependents not only on the existing body of medical knowledge, but also on available resources and specific case data. As argued by Yao and Kumar [59], physicians with different skill sets and fields of expertise may offer differing care plans to the same patient. Additionally, as physicians handle a lot of cases each day, they are prone to making mistakes in performing procedures and making diagnoses [59]. Thus, the formal description of CP models adopted to the phases of their implementation would minimize the likelihood of the mistakes and optimize the work of the health professionals. 

Having said that, it should be noticed that the lack of a unified standard of description for CPs results in severe problems with ongoing cooperation and dissemination of knowledge between different healthcare units. The problems arise not only due to the large number of different forms of description for CPs, but also the variability in the detail of description. In consequence, transparency and the ease of exchanging and using CPs resulting therefrom, are minuscule, due to the form of presentation. Furthermore, a considerable number of clinical pathway descriptions contain elements from local document templates, which enable the user to enter performance data (drop down fields, input fields with patient data, confirmation of performance, results of medical procedures, etc.). This has resulted in further complication, which in turn, has led to a severe lack of transparency of the clinical pathway documents (Figure 3 is a good example).

At the same time, this approach limited the functionality of CPs to very elaborate ITPs for specific patients (third level clinical pathway), and their recipients, to physicians, sometimes including other medical personnel involved in the treatment process. This form makes it practically impossible to use CPs to facilitate communication between the patient and their closest relatives, or to facilitate education. It also hinders the use of CPs to disseminate and verify medical knowledge, by and between, different healthcare units. The lack of unified rules of description means that the evaluation of each clinical pathway must inevitably start with trying to get to terms with the specific “notation” itself, which has been used in each particular context [59].

With the increasing adaptation of artificial intelligence and medical robots, it is becoming increasingly more common for descriptions of elements of CPs to include algorithms and programs dedicated thereto. At the same time, CPs are increasingly becoming the primary source of data for devices that support the physician and other medical personnel such as mobile application, virtual assistant [12,61].

The search for definitions and methods of describing CPs is ongoing, with relevant literature proposing a broad range of forms of description [44,58,59,60]. The most common of these are: verbal description, structured description (usually, in accordance with binding models and procedures); block diagram; tables; checklists; process diagrams; Gantt diagrams; 3D process maps, or the combination of all eight within one document [60,62].

### 2.4. Proposal of a Process-Based Approach to Describing Clinical Pathways

Process management has been faced with similar issues in the past. Going from written procedures and flowcharts, to drawing out entire detailed business processes in the form of a single diagram, has resulted in the end result being:

- Cumbersome (a size of even A0, i.e., 1 meter in width and height!);
- Cluttered (a large number of small objects and a plethora of interconnections);
- Too complex (includes a large amount of information, which is not pertinent to the specific patient).

These issues were solved by developing:

- Standard procedures dividing processes into hierarchical levels: maps, process models, and action charts with information, with the adequate level of detail and adequate scope of subject matter [61];
- Standard goals and rules of dividing the entire process into sub-processes on specific levels, and a clear presentation of their interrelations [61];
- Notation of description of processes, independent of the country in question, the geographical area, and the IT tool used to model processes (e.g., Business Process Model and Notation—BPMN) [42].

The issues with describing CPs discussed in the previous sections result from the lack of consequence in using methodologies and process tools, when dealing with diagnostic and therapeutic processes, which CPs are. In order to avoid such issues, descriptions of CPs should be prepared with the use of principles, methodologies, and notations, commonly and successfully adapted in process management.

It is essential to adapt the modeling language to the specifics of a given field, as well as to the level and scope of competence of the recipients and users of the prepared models [63,64]. For multiple uses (as in corporate architecture), separate description views and process models should be prepared with specific groups of users in mind, which should only contain crucial information and forms of description dedicated to a particular group, with a view to raising transparency and focus [65]. After all, process descriptions and models are not prepared for the modelers themselves, but rather, for a much broader group of recipients, who will use them in their daily work and volunteer improvements. In effect, it would be unwise to focus on the habits and views of a narrow group of ‘process experts’ in the selection of a notation for process description. Such an approach runs the risk of the repository of knowledge on processes becoming useless due to the illegible nature of the information contained therein, for the broader personnel in the organization [9]. For this reason, the intelligibility and legibility of descriptions of CPs is also a crucial value for the healthcare sector, because it has a direct impact on the effects of their use: on the effectiveness of therapy, and on the economic efficiency of health care units. The significance of this topic may be attested to by the results of a study held by the Centre for Policy on Ageing (CPA) [66]. In studies of various aspects of the use of CPs, on average, in 65% of cases, their positive influence on increasing the effectiveness of therapy and reducing the number of complications was apparent, whilst over 82% indicated their positive influence in terms of limiting the time of hospitalization and reducing costs. In the study held by Price Waterhouse Coopers [67], almost 700 leaders from the healthcare sector from 27 countries from all over the world were asked about their opinions on the significance of the transparency of information on quality, and prices for the sustainable healthcare system. Almost half of the leaders in question answered that this aspect is “very important” [67].

## 3. Materials and Methods 

In order to verify the need for variability in the presentation of CPs in various phases of their implementation, and to define the forms of presentation of CPs required by physicians in each phase, an exploratory pilot survey in a large multi-specialization hospital in Poland was conducted as it allowed for gathering the opinions of physicians from many specializations, and thus, with many different points of view. Exploratory survey was chosen as the study is one of the first attempts to establish the CPs forms and factors determining the choice of clinical pathway presentation in HIS/EMR systems.

The St. Padre Pio Provincial Hospital in Przemyśl was chosen for its readiness to participate in the research, which engages a number of physicians for a relatively long period of time. The survey sample was informative and consisted of purposely selected heads of all hospital departments and the medical team of the pediatric ward (*n* = 28). The sample size is appropriate, as Isaac and Michael [68] and Hill [69] suggested 10 to 30 participants for a pilot survey research.

In order to establish the conceptual framework of the questionnaire handed to each study participant, the fundamental terms were defined: business process, diagnostic–therapeutic process, clinical pathway, etc. Furthermore, all of the forms of descriptions appearing in the questionnaire were defined, and graphical examples thereof were provided. Prior to the questionnaire, all of the groups were invited to a short meeting, which explained in detail the aim of the study, as well as all of the terms used in the study, in the context of the daily work of a medical doctor.

The questionnaire covered two sections. The first section focused on the preferred forms of description for CPs, during three phases:

- Patient diagnosis and the formulation of a treatment plan (patient diagnosis; preparation of an individual treatment plan (ITP);
- Patient treatment, including eventual modifications to the ITP (modification of the individual treatment plan; adding a step to the individual treatment plan; preparation of a medical treatment/procedure; reminder on the necessity of performing a step of the individual treatment plan; confirmation of the performance of a step of the individual treatment plan; analysis of the performed diagnostic–therapeutic process);
- Ex-post evaluation of the finished treatment (analysis of the course of a finished diagnostic–therapeutic process of an individual patient; comparative analysis of a finished diagnostic–therapeutic process and the clinical pathway; comparative analysis of multiple finished diagnostic–therapeutic processes and the clinical pathway; statistical analysis of finished diagnostic–therapeutic processes; statistical analysis of finished diagnostic–therapeutic processes).

For each phase of the treatment process, eight possible forms of CP descriptions were proposed (verbal description, structured description, block diagram, table, checklist, process diagram, Gantt diagram, and 3D process map) [70,71,72,73]. 

The second section contains questions designed to get information about the key factors behind the choice of a particular form of description of CP, divided as before into three phases (diagnosis and planning, treatment, and ex-post evaluation). 

In both instances, for each position representing key preferences or determinants selected by the doctors, for particular phases of the treatment process, the participants were allowed to select up to three possibilities from seven predefined ones:

- Clearer form of patient data, making it easier to make correct clinical decisions;
- Possibility of evaluating the treatment on an ongoing basis;
- Possibility of modifying planned ongoing actions on an ongoing basis;
- Possibility of analyzing use resources and the degree of their productivity;
- Limiting mistakes in treatment by monitoring;
- Possibility of easier comparative analysis with the use of anonymized data on the treatment of other patients;
- Better control over the operations of the organization.

Respondents could assign one of the three values to each: 3, 2, or 1, respectively, where 3 meant the most preferred value, and 1 meant the least preferred of the three.

## 4. Results

Results of the study on the forms of descriptions of CPs preferred by physicians are presented in Table 2. The numbers presented in the table constitute the arithmetic sum given to the given vote option. 

As the study has demonstrated, most physicians prefer the preparation of an individual treatment plan (ITP) in the form of a checklist (19 votes from 42 votes cast, which gives more than 45%). At the same time, 50% of the respondents prefer a modification of the ITP in the form of a checklist, while fewer than 16% prefer the CP in the form of a process diagram. None of the physicians have indicated the possibility of preparing an ITP in the form of a Gantt diagram, and in the treatment phase, as few as 4% of the respondents, considered working with a form of description of a CPs in the form of a Gantt diagram (only in case of the analysis of the performed diagnostic–therapeutic process).

In the ex-post evaluation phase, most of the respondents (31%) selected the Gantt diagram and only 18% of the respondents opted for a process diagram. Surprisingly, 17% of physicians taking part in the study opted for the 3D process map—the form that was ignored in the first two phases. The verbal description is not considered as the form of presentation of CPs at this phase. 

Regardless of the detailed relationship between preferences for individual forms of CPs presentation, the results of this part of the study clearly indicate the need for variability in the presentation of CPs, in various phases of their implementation. As presented in Table 2, each phase or group of performed actions reveal the preference of 1–3 forms of presentation. The results should indicate for vendors of HIS and EMR the need to expand the systems and make this functionality available.

In the second part of the study, the physicians indicated which factors influenced the choice of their preferred form of CPs (Table 3). In the treatment planning phase, the main determinants are ‘clearer form of patient data, making it easier to make correct clinical decisions’ and ‘possibility of evaluating the treatment on an ongoing basis’. Furthermore, the ‘possibility of modifying planned and ongoing actions on an ongoing basis’ is considered as an important factor. In the treatment phase the most important determinants are again ‘possibility of evaluating the treatment on an ongoing basis’ (30%), ‘possibility of modifying planned and ongoing actions on an ongoing basis’ (26%), and ‘clearer form of patient data, making it easier to make correct clinical decisions’ (21%). However, in the ex-post evaluation phase the key determinants are not only ‘possibility of evaluating the treatment on an ongoing basis’ (23%) and ‘possibility of modifying planned and ongoing actions on an ongoing basis’ (20%), but also ‘possibility of analyzing used resources and the degree of their productivity’ and ‘limiting mistakes in treatment by monitoring’—16% each. 

In all of the phases—planning, treatment, and ex-post evaluation of CPs—the main determinant (or one of the main determinants) of their choice was ‘possibility of evaluating the treatment on an ongoing basis’ (27%) and ‘possibility of modifying planned and ongoing actions on an ongoing basis’ (23%).

## 5. Discussion

In conclusion, the assumption that the form of presentation of a business process should remain unchanged throughout the entire process of execution of phases is false. The results of the study clearly demonstrate the variability of the physicians’ expectations toward the form of description of the diagnostic–therapeutic process (Table 4). This is both a consequence of the different goals of using a process description in different phases, as well as from clearly different contexts in which process performers operate.

In the initial diagnosis and treatment phase, physicians have no direct time constraints and have the opportunity to consult and modify treatment plans. That is why they prefer more general forms of CP presentations like checklists or structured descriptions allowing for the flexibility. However, in the patient treatment phase respondents experience strong time constrains and pressure of carried responsibility as the undertaken actions are often irreversible. Thus, in this phase, physicians more often opt for block diagrams and process diagrams, but still physicians prefer process descriptions in the form of a checklist, which facilitates the ongoing evaluation of treatment and communication as part of the therapy team thanks to its transparent, intuitive form of presenting information [35]. Checklists not only facilitate the making of correct clinical decisions, but also can be modified and easily developed with new tasks [74,75].

In the ex-post evaluation phase, physicians operate without time pressure as the patient is in stable condition. The key task is to identify the potential threats which could impact the patient’s further treatment. Additional objective is also the evaluation of current knowledge and, if possible, the identification of new knowledge, which stems from the diagnostic–therapeutic process. The description of processes, supplemented with parameters defining the patient’s health, enables physicians to make an in-depth evaluation of the performed actions and their results within the framework of the individual treatment process. For example, a 3D process map allows for the graphical comparative evaluation of a clinical pathway, an ongoing or completed treatment process for an individual patient, and multiple different treatments, data on which is stored in Evidence Based Medicine (EBM) databases [35].

An important limitation of the research, resulting directly from its goals, is to focus only on the form and determinants of the CPs presentation omitting aspects related to the collection of data on the implemented diagnostic and therapeutic processes, and verification and updating of CPs based on the collected data. This is the natural direction of further CPs research as kiBPs, enabling the disclosure of tacit knowledge created in process execution.

## 6. Conclusions

The aim of the paper was to indicate the need for variability in the presentation of CPs in various phases of their implementation, and define the forms of presentation of CPs required by physicians in HIS/EMR systems, as well as the determinants of the selection of the chosen forms of description, in relation to the performed phases of therapy. 

The results of the study have unambiguously supported the claim that physicians require the form of presentation of CPs to change, depending on the particular phase of the diagnostic–therapeutic process, as well as establishing the main determinants thereof.

According to the study’s results, regardless of the phase of the CPs, the most important factors determining the choice of the form of description of a CP, preferred by the physicians, are:

- The possibility to analyze the treatment on an ongoing basis (27%);
- The possibility to dynamically modify planned and ongoing actions (23%).

Together, both determinants comprise 50% of the preferences. Their correspondence with the third and the second principles of dynamic BPM, respectively, conclusively points to the need to dynamically manage CPs. At the same time, it points to the necessity of conducting further studies on CPs as diagnostic–therapeutic processes, whose results will be an important contribution to the theory and research on the practical implications of dynamic BPM.

### 6.1. Theoretical Contribution

The authors believe that the study makes a significant contribution to the existing literature. The results of the study are, to the authors’ knowledge, the first that present in-depth analysis of the determinants of CPs description from the process management approach perspective. From the theoretical perspective, the paper presents forms of the CPs descriptions, and the possible process-centered approach application. The results of the study indicate that physicians generally require adapting the form of CPs to the phase of the executed diagnostic–therapeutic process and to enable the individualization of the form of CPs presentation, and thus the individualization of the form of presentation and data input during the therapy.

### 6.2. Practical Implication

The results of the study, the authors’ own experiences with consulting projects, and similar experiences of other scholars (e.g., [28,35,75]), demonstrate the belief that the method of presenting, as well as the modification of the description of dynamically managed business processes should change, according to:

1. The level of description;
2. The character of the processes (the field that is being modeled), and the group of recipients; but also
3. The phases of the process execution.

Satisfying conditions 2 and 3 simultaneously is impossible without including in IT systems supporting business process execution (e.g., diagnostic–therapeutic processes execution), the possibility of flexibly re-defining, as well as creating different process views ad hoc, depending on user needs [8,28,29,62,75]. Elements available in a given process view and their degree of detail are, of course, dependent on the tasks that particular participants of the process face at a specific phases of the process and the opportunities arising from the available equipment and competence of the health care unit’s staff. The available views should be tailored to the individual habits, needs, and limitations of the users, stemming from—e.g., their performed tasks. At the same time, the scope of possibilities resulting from the available equipment and the competences of the staff should be defined using generally accepted, well-known, and clear terminology enabling the flow of information within the health care unit and between health care units, e.g., SNOMED-CT. This will enable rapid updating and extensive communication of knowledge contained in CPs through:

- Updating the medical knowledge contained in them based on the latest achievements of medicine, passing from Clinical Guidelines for Individual Treatment Plan (ITP) [12];
- Updating information on medical procedures that can be implemented within the health care unit, based on the common ontology underlying the CPs;
- Accumulation of knowledge on the basis of implemented or completed diagnostic and therapeutic processes thanks to the use of process mining techniques and the collection of data on clinical decisions taken and their impact on the course and results of therapy.

### 6.3. Limitations with Future Research Directions

Having said that, it must be highlighted that the research results have certain limitations, as this is only a pilot study, and the sample is small and refers only to one Polish hospital. Second, the diversification of the diagnostic and therapeutic processes is also problematic for the assessment of the determinants of the form of description of CPs. Third, as we are drawing from ad hoc research, we present a snapshot of the physicians’ preferences, and the analysis does not reveal changes in those preferences over time, due to technological changes, the skills of new generations of physicians, etc.

The natural direction for further research would be as follows:

1. Analysis of preferences of users of CP presentation form conducted on a wide group of their users (doctors, nurses and other medical personnel);
2. The identification of determinants for the description and presentation of CPs in different phases of their execution, on stationary and mobile devices;
3. Development of a standard CPs integration format with ontologies describing resources and possible medical procedures, as well as knowledge bases or Evidence Based Medicine (EBM);
4. The preparation of guidelines, with respect to the user interface for creators of HIS/EMR systems, as well as, more generally, creators of IT systems supporting dynamic BPM;
5. Development and practical verification of the methodology and tools for gathering knowledge based on the implemented CPs and its use for ongoing support of the doctor’s work in the field of clinical decision-making.

In future research, it would be also interesting to analyze the determinants of the forms of descriptions of CPs, focused on updating critical success factors (CSF), and their use, not just within the healthcare unit, but also, in accordance with the first principle of dynamic BPM, first and foremost, within a holistic view of patient treatment, within a broadly understood healthcare eco-system.

## Figures and Tables

**Table 1:** Examples of mutually exclusive expectations for the descriptions of diagnostic–therapeutic processes.

Administration and Supervision Priorities	Physicians’ Requirements (Expectations)
The standardization of diagnostic and therapeutic processes on the basis of acquired knowledge.	Adapting such processes to the situation at hand and obtaining new knowledge from each subsequent performance.
Planning and strict control over performance.	Empowering physicians to make independent clinical decisions.
Cost optimization of the performed processes.	Allowing for the accommodation of a given performance to the needs of the individual patient.

**Table 2:** Forms of description of CPs preferred by physicians.

Form of Description and Presentation of CPs/Phase or Group of Performed Actions	Verbal Description	Structured Description	Block Diagram	Table	Checklist	Process Diagram	Gantt Diagram	3D Process Map	Total No. Points
patient diagnosis	2	7	4	6	18	6	0	1	*44*
preparation of an individual treatment plan (ITP)	3	6	5	4	19	2	0	3	*42*
**Total: The form of description of CPs in the initial diagnosis and treatment planning phase**	**5**	**13**	**9**	**10**	**37**	**8**	**0**	**4**	**86**
	*6%*	*15%*	*10%*	*12%*	*43%*	*9%*	*0%*	*5%*	
modification of the individual treatment plan	2	3	3	3	16	5	0	0	*32*
adding a step to the individual treatment plan	9	6	4	7	11	7	0	0	*44*
preparation of a medical treatment/procedure	0	6	12	3	12	11	0	0	*44*
reminder on the necessity of performing a step of the individual treatment plan	6	11	9	3	10	3	0	0	*42*
confirmation of the performance of a step of the individual treatment plan	6	11	5	3	9	8	0	0	*42*
analysis of the performed diagnostic-therapeutic process	0	10	3	7	3	3	10	2	*38*
**Total: The form of decription of CPs in the patient treatment phase**	**23**	**47**	**36**	**26**	**61**	**37**	**10**	**2**	**242**
	*10%*	*19%*	*14.9%*	*11%*	*25%*	*15.3%*	*4%*	*1%*	
analysis of the course of a finished diagnostic-therapeutic process of an individual patient	0	0	3	6	7	5	11	9	*41*
comparative analysis of a finished diagnostic-therapeutic process and the clinical pathway	0	2	0	6	5	5	12	8	*38*
comparative analysis of multiple finished diagnostic-therapeutic processes and the clinical pathway	0	2	2	8	2	10	11	3	*38*
statistical analysis of finished diagnostic-therapeutic processes	0	5	0	4	2	7	12	8	*38*
statistical analysis of finished diagnostic-therapeutic processes	0	5	0	1	5	7	12	5	*35*
**Total: The form of description of CPs in the ex-post evaluation phase**	**0**	**14**	**5**	**25**	**21**	**34**	**58**	**33**	**190**
	*0%*	*7%*	*3%*	*13%*	*11%*	*18%*	*31%*	*17%*	
**Total: The form of description of CPs expected by physicians**	**28**	**74**	**50**	**61**	**119**	**79**	**68**	**39**	**518**
	*5%*	*14%*	*10%*	*12%*	*23%*	*15%*	*13%*	*8%*	

**Table 3:** Determinants of the choice of the form of description of CPs preferred by physicians.

Determinants of the Selection of the Form of Description of CPs/Phase or Group of Performed Actions	Clearer Form of Patient Data, Making it Easier to Make Correct Clinical Decisions	Possibility of Evaluating the Treatment on an Ongoing Basis	Possibility of Modifying Planned and Ongoing Actions on an Ongoing Basis	Possibility of Analysing Used Resources and the Degree of Their Productivity	Limiting Mistakes in Treatment by Monitoring	Possibility of Easier Comparative Analysis with the Use of Anonymized Data on the Treatment of Other Patients	Better Control over the Operations of the Organization	Total No. Points
patient diagnosis	9	9	4	0	1	0	2	25
preparation of an individual treatment plan	12	12	8	3	1	0	3	39
Total: The form of description of CPs in the initial diagnosis and treatment planning phase	21	21	12	3	2	0	5	64
	33%	33%	19%	5%	3%	0%	8%	
modification of the individual treatment plan	10	11	14	0	4	0	3	42
adding a step to the individual treatment plan	10	9	12	0	2	1	2	36
preparation of a medical treatment/procedure	9	11	9	0	7	2	2	40
reminder on the necessity of performing a step of the individual treatment plan	7	11	10	0	9	0	1	38
confirmation of the performance of a step of the individual treatment plan	10	14	6	2	3	1	2	38
analysis of the performed diagnostic-therapeutic process	3	14	10	4	2	3	6	42
Total: The form of decription of CPs in the patient treatment phase	49	70	61	6	27	7	16	236
	21%	30%	26%	3%	11%	3%	7%	
analysis of the course of a finished diagnostic-therapeutic process of an individual patient	0	9	9	6	5	3	7	39
comparative analysis of a finished diagnostic-therapeutic process and the clinical pathway	0	6	6	7	7	7	3	36
comparative analysis of multiple finished diagnostic-therapeutic processes and the clinical pathway	0	9	9	6	6	6	3	39
statistical analysis of finished diagnostic-therapeutic processes	0	9	7	4	7	5	4	36
statistical analysis of finished diagnostic-therapeutic processes	2	9	6	6	5	5	3	36
Total: The form of description of CPs in the ex-post evaluation phase	2	42	37	29	30	26	20	186
	1%	23%	20%	16%	16%	14%	11%	
Total: The form of description of CPs expected by physicians	72	133	110	38	59	33	41	486
	15%	27%	23%	8%	12%	7%	8%	

**Table 4:** Variability of the main features of the context of performance, and the preferred forms of descriptions, at different phases of the CPs.

Phase of the CP Lifecycle		Main Features of the Context of Executing the CP	The Preferred Form of Description of the CP	Determinants of the Choice of the Preferred Form of the CP Description and Presentation by Physicians
I	Initial diagnosis and treatment planning	Usually the lack of direct time constraints. The possibility of consulting and modifying treatment plans multiple times.	1. Checklist 2. Structured description 3. Table	1. The possibility of analyzing the treatment on an ongoing basis. 2. Clearer form of patient data, making it easier to make correct clinical decisions. 3. The possibility of dynamically modifying planned and ongoing actions.
II	Patient treatment	Time constraints (or very strong time constraints). The necessity of tailoring the prepare treatment plan to the course of a specific treatment, including unpredictable developments. Responsibility (the undertaken actions are often irreversible).	1. Checklist 2. Structured description 3. Process diagram	1. The possibility of analyzing the treatment on an ongoing basis. 2. The possibility of dynamically modifying planned and ongoing actions. 3. Clearer form of patient data, making it easier to make correct clinical decisions.
III	Ex-post evaluation of finished treatments	No time constraints. The possibilit of consulting and modifying or supplementing the results of analyses multiple times.	1. Gantt diagram 2. Process diagram 3. Table	1. The possibility of analyzing the treatment on an ongoing basis. 2. The possibility of dynamically modifying planned and ongoing actions. 3. The possibility of analysing used resources and the degree of their productivity

## References

[1] Puah P., Tang N. Business process management, a consolidation of BPR and TQM. Proceedings of the 2008 IEEE Conference on Cybernetics and Intelligent Systems.

[2] Khan R.N. (2004). Business Process Management: A Practical Guide.

[3] Chang J.F. (2006). Business Process Management Systems: Strategy and Implementation.

[4] Jeston J., Nelis J. (2013). Business Process Management. Practical Guidelines to Successful Implementations.

[5] Dumas M., La Rosa M., Mendling J., Reijers H. (2016). Fundamentals of Business Process Management.

[6] van Herck P., Vanhaecht K., Sermeus W. (2004). Effects of Clinical Pathways: Do they work?. Int. J. Care Pathw..

[7] Hindle D., Dowdeswell B., Yasbeck A. (2004). Report of a Survey of Clinical Pathways and Strategic Asset Planning in 17 EU Countries.

[8] Rotter T., Kinsman L., James E., Machotta A., Gothe H., Willis J., Snow P., Kugler J. (2010). Clinical Pathways: Effects on Professional Practice, Patient Outcomes, Length of Stay and Hospital Costs. Cochrane Database Syst. Rev..

[9] Kirchmer M., Laengle S., Masias V. (2013). Transparency-Driven Business Process Management in Healthcare Settings. IEEE Technol. Soc. Mag..

[10] Hellman S., Kastberg G., Siverbo S. (2015). Explaining process orientation failure and success in health care—Three case studies. J. Health Organ. Manag..

[11] Szelągowski M. (2015). Nowe metody zarządzania procesowego w ochronie zdrowia. e-Mentor.

[12] Marrella M., Mecella M., Sharf M., Catarci T. The TESTMED Project Experience Process-aware Enactment of Clinical Guidelines through Multimodal Interfaces. https://arxiv.org/pdf/1807.02022.pdf.

[13] Rummler G., Brache A. (2000). Podnoszenie Efektywności Organizacji (Improving Performance).

[14] Knudson G. What Is BPM?. http://www.bpmleader.com/2013/07/29/what-is-bpm/.

[15] Campbell H., Hotchkiss R., Bradshaw N., Porteous M. (1998). Integrated care pathways. Br. Med. J..

[16] González Sánchez M.J., Framiñán Torres J.M., Parra Calderón C.L., Del Río Ortega J.A., Vigil Martín E., Nieto Cervera J. (2008). Application of business process management to drive the deployment of a speech recognition system in a healthcare organization. Stud. Health Technol. Inform..

[17] Andellini M., Fernandez Riesgo S., Morolli F., Ritrovato M., Cosoli P., Petruzzellis S., Rosso N. (2017). Experimental application of Business Process Management technology to manage clinical pathways: A pediatric kidney transplantation follow up case. BMC Med. Inform. Decis. Mak..

[18] Scheuerlein H., Rauchfuss F., Dittmar Y., Molle R., Lehmann T., Pienkos N., Settmacher U. (2012). New methods for clinical pathways—Business Process Modeling Notation (BPMN) and Tangible Business Process Modeling (t.BPM). Langenbeck’s Arch. Surg..

[19] Taylor F.W. (1911). The Principles of Scientific Management.

[20] Zairi M. (1997). Business process management: A boundaryless approach to modern competitiveness. Bus. Process Manag. J..

[21] Palmberg K. (2009). Exploring process management: Are there any widespread models and definitions?. TQM J..

[22] Röglinger M., Pöppelbuß J., Becker J. (2012). Maturity Models in Business Process Management. Bus. Process Manag. J..

[23] Kania K. (2013). Doskonalenie Zarządzania Procesami Biznesowymi w Organizacji z Wykorzystaniem Modeli Dojrzałości i Technologii Informacyjno-Komunikacyjnych.

[24] Porter M. (1985). Competitive Advantazge.

[25] Davenport T., Short J. (1990). The new industrial engineering: Information technology and business process redesign. Sloan Manag. Rev..

[26] Richter-von Hagen C., Ratz D., Povalej R. (2005). Towards Self-Organizing Knowledge Intensive Processes. J. Univers. Knowl. Manag..

[27] Di Ciccio C., Marrella A., Russo A. Knowledge-intensive Processes: An Overview of Contemporary Approaches?. Proceedings of the First International Workshop on Knowledge-Intensive Business Processes (KiBP 2012).

[28] Swenson K. (2010). Mastering the Unpredictable: How Adaptive Case Management Will Revolutionize the Way That Knowledge Workers Get Things Done.

[29] Szelągowski M. (2014). Konsekwencje dynamic BPM. e-Mentor.

[30] Gartner IT Glossary Dynamic Business Process Management. http://www.gartner.com/it-glossary/dynamic-business-process-management-bpm.

[31] Kemsley S., Rinderle-Ma S., Toumani F., Wolf K. (2011). The Changing Nature of Work: From Structured to Unstructured, from Controlled to Social. Business Process Management (BPM).

[32] Pucher M., Swenson K., Palmer N. (2012). How to link BPM governance and social collaboration through an Adaptive Paradigm. Social BPM: Work, Planning and Collaboration under the Impact of Social Technology.

[33] Rothschadl T., Stary C. (2012). Ad-hoc adaptation of subject-oriented business processes at runtime to support organizational learning. S-BPM ONE—Scientific Research: 4th International Conference, S-BPM ONE 2012.

[34] Kemsley S. (2010). Runtime collaboration and dynamic modeling in BPM: Allowing the Business to shape its own processes on the fly. Cut. IT J..

[35] Szelągowski M. (2019). Dynamic BPM in the Knowledge Economy: Creating Value from Intellectual Capital.

[36] Czekaj J. (2009). Metody Zarządzania Procesami w Świetle Studiów i Badań Empirycznych.

[37] Gronau N., Müller C., Korf R. (2005). KMDL—Capturing, Analysing and Improving Knowledge-Intensive Business Processes. J. Univers. Comput. Sci..

[38] Di Ciccio C., Marrella A., Russo A. (2015). Knowledge-intensive Processes Characteristics, Requirements and Analysis of Contemporary Approaches. J. Data Semant..

[39] Vanhaecht K., Ovretveit J., Elliott M.J., Sermeus W., Ellershaw J., Panella M. (2012). Have We Drawn the Wrong Conclusions About the Value of Care Pathways? Is a Cochrane Review Appropriate?. Eval. Health Prof..

[40] Davenport T., Nohria N. (1994). Case Management and the Integration of Labor. MIT Sloan Management Review Magazine.

[41] Zander K. (1988). Nursing case management: Strategic management of cost and quality outcomes. J. Nurs. Adm..

[42] OMG Business Process Model and Notation (BPMN). http://www.omg.org/spec/BPMN/2.0.2.

[43] Cheah T.S. (1998). Clinical Pathways—The New Paradigm in Healthcare?. Med. J. Malays..

[44] De Bleser L., Depreitere R., De Waele K., Vanhaecgt K., Vlayen J., Sermus W. (2006). Defining pathways. J. Nurs. Manag..

[45] Kitchiner D., Bundred P. (1996). Integrated care pathways. Arch. Dis. Child..

[46] Kononowicz A., Sałapa K. (2011). Ścieżki kliniczne. Elementy Informatyki Medycznej.

[47] Williams S., Radnor Z. (2017). An integrative approach to improving patient care pathways. Int. J. Health Care Qual. Assur..

[48] European Pathway Association (EPA) Care Pathways. http://e-p-a.org/care-pathways/.

[49] Vanhaecht K. (2007). The Impact of Clinical Pathways on the Organisation of Care Processes. Ph.D. Dissertation.

[50] Vanhaecht K., Panella M., van Zelm R., Sermeus W. (2010). An overview on the history and concept of care pathways as complex interventions. Int. J. Care Pathw..

[51] Wolff A., Taylor S., McCabe J. (2004). Using checklists and reminders in clinical pathways to improve hospital inpatient care. Med. J. Aust..

[52] Panella M., Vanhaecht K. (2010). Is there still need for confusion about pathways?. Int. J. Care Pathw..

[53] Joint Commission on Accreditation of Health Care Organizations Root Causes for Sentinel Events. http://www.jointcommission.org/assets/1/18/Root_Causes_Event_Type_2004-3Q2011.pdf.

[54] Kononowicz A. (2011). System Wspomagania Nauczania Medycyny Oparty na Koncepcji Ścieżek Klinicznych. Ph.D. Dissertation.

[55] Zander K. (2002). Integrated care pathways: Eleven international trends. Int. J. Care Pathw..

[56] Jung J., Choi I., Song M. (2007). An integration architecture for knowledge management systems and business process management systems. Comput. Ind..

[57] Schrijvers G. (2016). Integrated care. Better and Cheaper.

[58] Cheah T.S. (1998). The impact of clinical guidelines and clinical pathways on medical practice: Effectiveness and medico-legal aspects. Ann. Acad. Med..

[59] Yao W., Kumar A. (2012). CONFlexFlow: Integrating flexible clinical pathways into clinical decision support systems using context and rules. Decis. Support Syst..

[60] State of Queensland Possible Cardiac Chest Pain Clinical Pathway. https://www.health.qld.gov.au/__data/assets/pdf_file/0021/439131/sw574-chest-pain-pathway.pdf.

[61] Nuance Voice-Enabled Virtual Assistants for Healthcare. https://www.nuance.com/healthcare/ambient-clinical-intelligence/virtual-assistants.html#video.

[62] Browning T.R. (2010). On the alignment of the purposes and views of process models in project management. J. Oper. Manag..

[63] Braun R., Schlieter H., Burwitz M., Esswein W. BPMN4CP Revised—Extending BPMN for Multi-Perspective Modeling of Clinical Pathways. Proceedings of the 49th Hawaii International Conference on System Sciences (HICSS 2016).

[64] Cardoso E., Labunets K., Dalpiaz F., Mylopoulos J., Giorgini P., Wang Ling T., Zeng Z., Li Lee M., Ngoc Le T. (2016). Modeling Structured and Unstructured Processes: An Empirical Evaluation. Improving the Correctness of Some Database Research Using ORA-Semantics.

[65] Sobczak A. (2013). Architektura korporacyjna. Aspekty Teoretyczne i Wybrane Zastosowania Praktyczne.

[66] Centre for Policy on Ageing The Effectiveness of Care Pathways in Health and Social Care. http://www.cpa.org.uk/information/reviews/CPA-Rapid-Review-Effectiveness-of-care-pathways.pdf.

[67] PricewaterhouseCoopers HealthCast 2020: Creating a Sustainable Future. https://www.pwc.com/il/he/publications/assets/2healthcast_2020.pdf.

[68] Isaac S., Michael W.B. (1995). Handbook in Research and Evaluation.

[69] Hill R. (1998). What sample size is “enough” in internet survey research. Interpers. Comput. Technol..

[70] Dufresne T., Martin J. Process Modeling for E-Business. INFS 770—Methods for Information Systems Engineering: Knowledge Management and E-Business. http://odesso.com/sites/default/files/documents/processmodeling.doc.

[71] Waszkowski R., Kiedrowicz M., Kubiak B., Maślankowski J. (2015). Business rules automation standards in business process management systems. Information Management in Practice.

[72] Betz S., Eichhorn D., Hickl S., Klink S., Koschmider A., Li Y., Trunko R. 3D Representation of Business Process Models. http://subs.emis.de/LNI/Proceedings/Proceedings141/gi-proc-141-005.pdf.

[73] Effinger P., La Rosa M., Soffer P. (2012). A 3D-Navigator for Business Process Models. Business Process Management Workshops (BPM).

[74] Gawande A. (2009). The Checklist Manifesto How to Get Things Right.

[75] Krogstie J., Sindre G., Jørgensen H. (2006). Process models representing knowledge for action: A revised quality framework. Eur. J. Inf. Syst..

